# Emerging trends and hot spots in autoimmune thyroiditis research from 2000 to 2022: A bibliometric analysis

**DOI:** 10.3389/fimmu.2022.953465

**Published:** 2022-08-11

**Authors:** Qiuxian Li, Wanyu Yang, Jiashu Li, Zhongyan Shan

**Affiliations:** Department of Endocrinology and Metabolism and the Institute of Endocrinology, National Health Commission (NHC) Key Laboratory of Diagnosis and Treatment of Thyroid Diseases, The First Hospital of China Medical University, China Medical University, Shenyang, China

**Keywords:** bibliometric analysis, autoimmune thyroiditis (AIT), Hashimoto thyroiditis, CiteSpace, VOSviewer

## Abstract

**Background:**

Autoimmune thyroiditis (AIT) is the most common autoimmune disease, affecting 3-5% patients worldwide. In recent years, approximately 200 articles on AIT have been published annually in various journals. However, to date, no article has systematically assessed the related literature. Therefore, we conducted a bibliometric analysis on AIT to reveal the dynamic scientific developments and help researchers gain a global perspective while exploring the hotspots and development trends.

**Methods:**

AIT-related articles and reviews from 2000 to 2022 were retrieved from the Web of Science Core Collection (WoSCC). The following search terms were used to extract document data: TS= (“ autoimmune thyroiditi*”) OR TI= (“chronic lymphocytic thyroiditi*”) OR TI=(hashimoto*) OR TI= (“postpartum thyroiditis”). We selected articles and reviews published in English from 2000 to 2022. Three software programs (VOSviewer, CiteSpace, Pajek) were employed to analyze the contribution and co-occurrence relationships of different references, countries/regions, institutes, journals and also keywords in this field.

**Results:**

This scientometric study included 2290 English papers published in 723 journals with 39661 co-cited references from 561 institutions in 120 countries/regions. Based on the reference and keyword analysis, researchers used to focus on “apoptosis”, “insulin resistance”, “encephalopathy”, “IFN-γ” related to AIT during the past 20 years. However, with the development of other novel directions such as “papillary thyroid cancer” (2018-2022), “Vitamin D” (2016-2022), “oxidative stress” (2018-2022), “polymorphism” (2019-2022) and “association” (2020-2022), researchers are more interested in the relationship between papillary thyroid carcinoma and AIT, the effect of vitamin D supplementation on AIT, the oxidative stress in thyroid disease as well as the influence of polymorphism.

**Conclusion:**

Bibliometric analysis of the outputs of AIT shows an overview of the current status of the research on AIT. The associations between papillary thyroid carcinoma, vitamin D, oxidative stress, polymorphism and AIT are major research frontiers. However, further research and collaboration are still required worldwide. Our findings can help researchers grasp the research status of AIT and quickly determine new directions for future research.

## Introduction

Autoimmune thyroiditis (AIT) is the most common autoimmune endocrine disease (1) and has an estimated prevalence of approximately 3-5% of the general population (2). AIT covers a wide spectrum of phenotypes, and Hashimoto thyroiditis (HT) which is a type of T-cell-mediated organ-specific chronic inflammation, is the classic form (3). In addition to this classic form, several other clinical pathologic entities are included under the term AIT: the fibrous variant, with glandular fibrosis and rapid progression toward hypothyroidism (4); the fibrous atrophic variant; Riedel’s thyroiditis; IgG4 thyroiditis; painless thyroiditis; postpartum thyroiditis; Hashimoto’s encephalopathy (5); the hashitoxicosis variant; and the juvenile form (6).

AIT predominantly affects women, and its incidence increases with age (7, 8). It was first described in 1912 (9) and was comparatively rare at that time (10). However, 110 years later, it has become the most common endocrine disorder (11, 12). Furthermore, it is also the most common cause of hypothyroidism (13). In the early stages of the disease, there are usually no clinical symptoms, but common symptoms of hypothyroidism, such as negative mood, anxiety, depression, dry skin, muscle cramps and fatigue, constipation, puffy eyes, cold intolerance, deep voice, slow thinking and poor memory, can develop gradually (14). Although the pathogenic mechanisms have not yet been completely defined, they are related to genetic influences, environmental triggers and epigenetic effects (15). AIT is well known to be associated with other autoimmune diseases, such as chronic autoimmune gastritis, vitiligo, rheumatoid arthritis, polymyalgia rheumatica, celiac disease, type 1 diabetes, Sjögren’s syndrome, systemic lupus erythematosus (SLE), multiple sclerosis, and sarcoidosis (16).

Over the past 110 years, a vast body of studies have been published concerning AIT. Such significant growth in the literature requires new approaches to review and analyze trends. However, there are no studies that systematically assess the related literature. CiteSpace and VOSviewer are bibliometric analysis software programs that use mathematical and statistical methods to quantitatively analyze a considerable number of documents in a specific domain, which can intuitively reveal the dynamic scientific development of cutting-edge knowledge, provide valuable help for researchers to compare the contributions of various institutions, countries, and journals, provide valuable references and guidance and encourage researchers to take a global view of their problems (17–21). The aim of this study is to compare the current development in this domain and explore the frontier issues of AIT from 2000 to 2022. The use of CiteSpace and VOSviewer software has helped us to better illustrate scientific knowledge and various interrelationships in order for researchers to better grasp current overall trends in this field.

## Material and methods

### Data source and search

The publications were obtained from the Core Collection database of Web of Science (WoSCC) because it is generally considered one of the most systematic, authoritative and comprehensive databases and has been widely used for scientometric analysis and visualization of scientific documents in a considerable number of studies (22–26). All searches were completed and downloaded on 1 day, March 20, 2022, to avoid the bias caused by daily database updates and verified by two authors independently. The following search terms were used to extract document data: TS= (“ autoimmune thyroiditi*”) OR TI= (“chronic lymphocytic thyroiditi*”) OR TI=(hashimoto*) OR TI= (“postpartum thyroiditis”). The selection criteria were as follows: (1) language: English; (2) document type: article or review; and (3) timespan: 2000–2022. Initially, there were 4845 documents, and 3599 satisfied these criteria. After screening each of these titles and abstracts, 2324 articles that exclusively targeted the topic of AIT were ultimately included. For the articles that satisfied these inclusion criteria, all records, including the titles, authors, abstracts, keywords and references, were exported, saved as plain text files and stored as download_txt files and then imported to CiteSpace5.8.R3. After using CiteSpace to analyze these documents, 2290 were subjected to visualization analysis.

### Data analysis and visualization

CiteSpace is a freely available Java application for the visualization and analysis of scientific literature trends and patterns (27). It was created by Professor Chaomei Chen in 2004 and has become popular globally to present the structure, laws, and distribution of scientific knowledge and provide a comprehensive overview of the research situation (28). Therefore, CiteSpace can be used to predict the research trend of a certain discipline or field during a certain period (29). The CiteSpace parameters were set as follows: link retaining factor (LRF = 3), e for top N (e = 2), time span (2000–2022), years per slice, look back years (LBY = 23), links (strength: cosine, scope: within slices), selection criteria (g-index: k = 25), and minimum duration (MD = 1) (30). VOSviewer is a program for building and viewing bibliometric maps (31). We used VOSviewer (version 1.6.18) to visualize the collaborations between countries, institutions, journals as well as high-frequency keywords. In VOSviewer, nodes were used to represent countries, institutions, journals or keywords, and the size of the node was determined by its frequency of co-occurrence in titles and abstracts (32). VOSviewer can be used to build journal maps based on collaborative data or to build keyword maps based on co-occurrence data (33). In the figure production process, we also used another software program, Pajek, an extremely professional web analytics tool, which was greatly beneficial for us to make the relationships clearer.

## Results

### Analysis of co-cited references: Clusters of research and most cited papers

**Figure 1:**
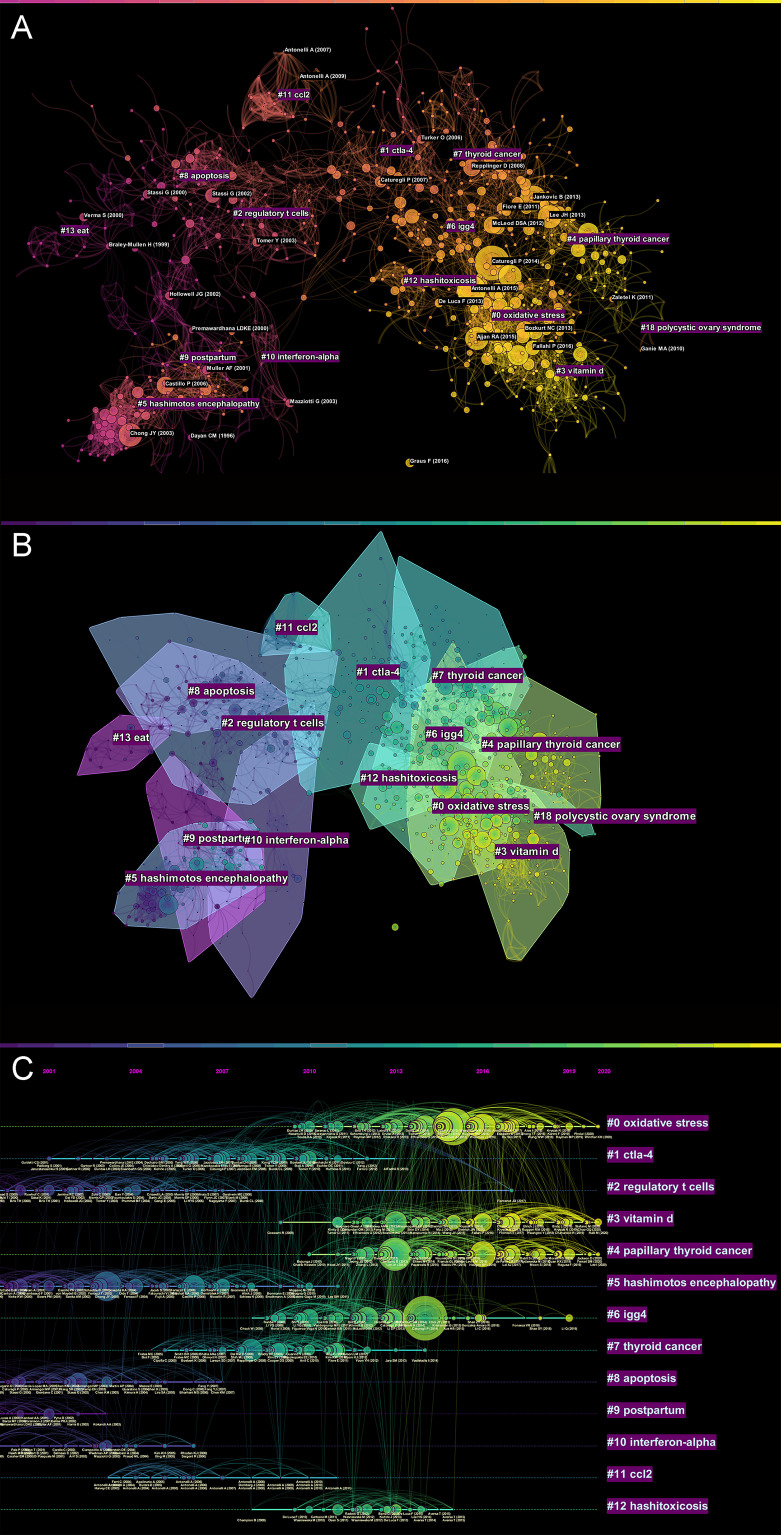
**(A)** Knowledge map of references related to AIT research. **(B)** The co-citation clusters of references related to AIT research. **(C)** The timeline view of references related to AIT research.

We used Citespace software to generate a map of reference co-citations with corresponding clusters so that we could extract landmark references and clusters of research (**Figure 1A**). Eighty-seven different clusters were identified in this network of co-citation references and we filtered the clusters which contained more than 10 documents, with significant modularity and silhouette scores (Q= 0.7964; S= 0.92). Q-values range from 0-1, with values greater than 0.3 indicating a significant delineation structure; S-values greater than 0.5 indicate reasonable clustering results, and 0.7 or more are more convincing.

When two publications are cited jointly by a third publication, this is referred to as a co-citation relationship (34). Therefore, co-cited references are usually regarded as a knowledge base in a particular field (35). The more frequently a piece of literature is cited, the more crucial it is considered in a certain field. We analyzed all of these documents, and a total of 1137 co-cited references were identified from 2000 to 2022 based on CiteSpace with the time slice set as a year. **Table 1** demonstrates the top 10 cited references. Each reference was co-cited at least 30 times. Fifty percent of the authors were from the United States. Among the top 10 cited references, the review entitled “*Hashimoto thyroiditis: clinical and diagnostic criteria”* published by P Caturegli et al. in the journal of *Autoimmunity review* was the most co-cited paper (n = 97), followed by *Autoimmune thyroid disorders*, which was also published in *Autoimmune thyroid disorders* (n=70). The remaining eight references had 30-44 co-citations. The literature entitled *Increased circulating pro-inflammatory cytokines and Th17 lymphocytes in Hashimoto’s thyroiditis* published in *The Journal of Clinical Endocrinology and Metabolism* had the largest centrality (0.16), indicating that it was the most acknowledged literature and had a significant influence on other researchers’ work.

**Table 1:** Top 10 co-cited references in the research of AIT.

Publication year	First author	Country/region	Title	Type	Journal	Citations	Centrality	2020 Impact factor
2014	P Caturegli (6)	USA	Hashimoto thyroiditis: clinical and diagnostic criteria	review	Autoimmunity Reviews	97	0.01	9.754
2015	Alessandro Antonelli	Italy	Autoimmune thyroid disorders	review	Autoimmunity Reviews	70	0.14	9.754
2015	R A Ajjan	UK	The Pathogenesis of Hashimoto’s Thyroiditis: Further Developments in our Understanding	review	Hormone and Metabolic Research	44	0.07	2.936
2003	Ji Y Chong	USA	Hashimoto encephalopathy: syndrome or myth?	review	Archives of neurology	44	0.01	7.419
2013	Ju-Han Lee (36)	Korea	The association between papillary thyroid carcinoma and histologically proven Hashimoto’s thyroiditis: a meta-analysis	meta-analysis	European journal of endocinology	43	0.05	6.664
2015	Aleksandra Pyzik (7)	Poland	Immune disorders in Hashimoto’s thyroiditis: what do we know so far?	review	Journal of immunology research	43	0.03	4.818
2013	Bojana Jankovic (37)	USA	Clinical Review: Hashimoto’s thyroiditis and papillary thyroid carcinoma: is there a correlation	review	The Journal of clinical endocrinology & metabolism	40	0.02	5.958
2012	Donald S A McLeod (2)	USA	The incidence and prevalence of thyroid autoimmunity	review	Endocrine	34	0.01	3.633
2016	Johanna Wichman (38)	USA	Selenium Supplementation Significantly Reduces Thyroid Autoantibody Levels in Patients with Chronic Autoimmune Thyroiditis: A Systematic Review and Meta-Analysis	review	Thyroid	31	0.03	6.568
2010	Nicté Figueroa-Vega	Spain	Increased circulating pro-inflammatory cytokines and Th17 lymphocytes in Hashimoto’s thyroiditis	review	The Journal of clinical endocrinology & metabolism	30	0.16	5.958

The top 11 co-citation clusters were shown in **Figure 1B**. In the timeline view, different colors of nodes on the same line indicated different years (**Figure 1C**) (30). Accordingly, the nodes that were closer to the right reflected the more recent references. CiteSpace was also a convenient tool for exploring the evolution track and stage characteristics of a specific research field. The clusters were listed from 1998 to 2016: “postpartum” (Cluster 9, n=54), “interferon-alpha” (Cluster 10 n=46), “apoptosis” (Cluster 8, n=59), “regulatory t cells” (Cluster 2, n=101), “hashimoto’s encephalopathy” (Cluster 5, n=95), “CCL2” (Cluster 11, n=26), “CTLA-4” (Cluster 1, n=107), “thyroid cancer” (Cluster 7, n=67), “IgG4” (Cluster 6, n=82), “oxidative stress” (Cluster 0, n=128), “papillary thyroid cancer” (Cluster 4 n=96), and “Vitamin D” (Cluster 3, n=98).

**Figure 2:**
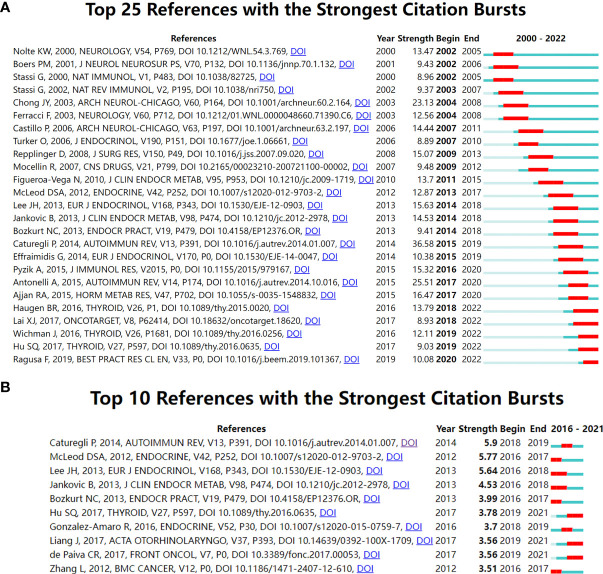
References with the strongest citation bursts related to AIT research. **(A)** Top 25 references with the strongest citation bursts related to AIT research during the past two decades. **(B)** Top 10 references with the strongest citation bursts related to AIT research in the past five years.

The designation “References with citation bursts” indicates that the relevant literature was cited at a high frequency during a certain period. The top 25 references with the strongest citation bursts are presented in **Figure 2A**. As illustrated, the blue line segment indicates the time interval, while the red line segment reflects the frequently cited time. This figure also indirectly reflected that the research in Hashimoto thyroiditis has a stable progression because each year has references with citation bursts, and many of them continue to 2022. The analysis of burstness revealed that the top 3 references with the strongest citation burst were *Hashimoto thyroiditis: Clinical and diagnostic criteria, Autoimmune thyroid disorders*, and *Hashimoto Encephalopathy: Syndrome or Myth.* When focusing on the last 5 years, as illustrated in **Figure 2B**, the top 3 references with the strongest citation burst were *Hashimoto thyroiditis: Clinical and diagnostic criteria*, *The incidence and prevalence of thyroid autoimmunity*, *The association between papillary thyroid carcinoma and histologically proven Hashimoto’s thyroiditis: a meta-analysis.*

### Annual growth trend

**Figure 3:**
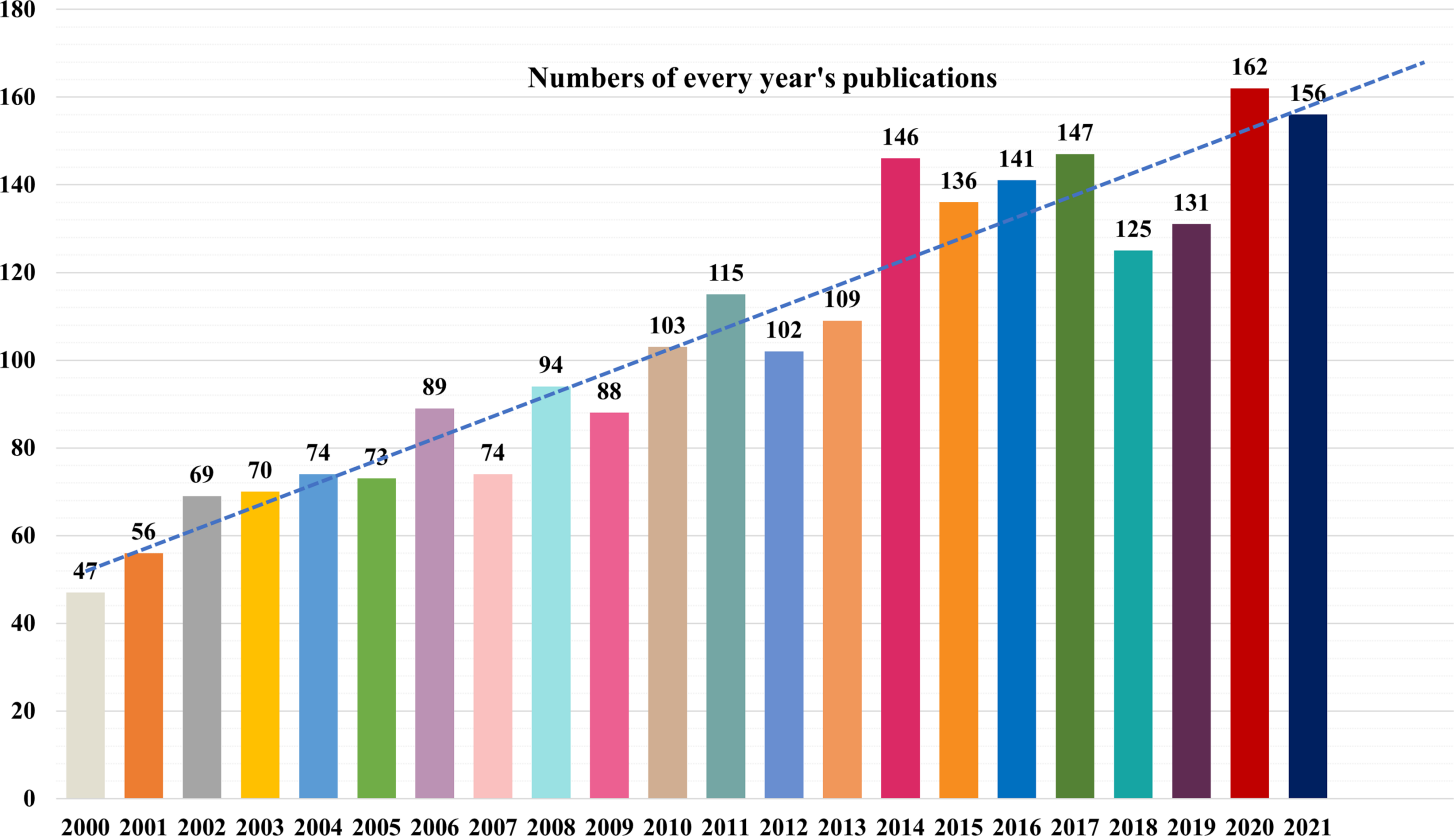
Trends in the number of publications concerning AIT and analysis of the associated countries/regions.

There were 2153 articles (92.64%) and 171 reviews (7.36%) among the 2324 documents, including 62 clinical trials, 28 RCT as well as 43 meta-analysis or systematic reviews. The distribution of publication number by year, presented in **Figure 3**, shows a relatively upward tendency, indicating a steady development of this field. The quantity of published documents demonstrates how quickly knowledge is updated in this subject and is an essential indicator for grasping the trends in this field. Only 47 publications about AIT were published in 2000, but 23 years later, the number of documents has approximately quadrupled.

### Distribution of countries or regions analysis

**Figure 4:**
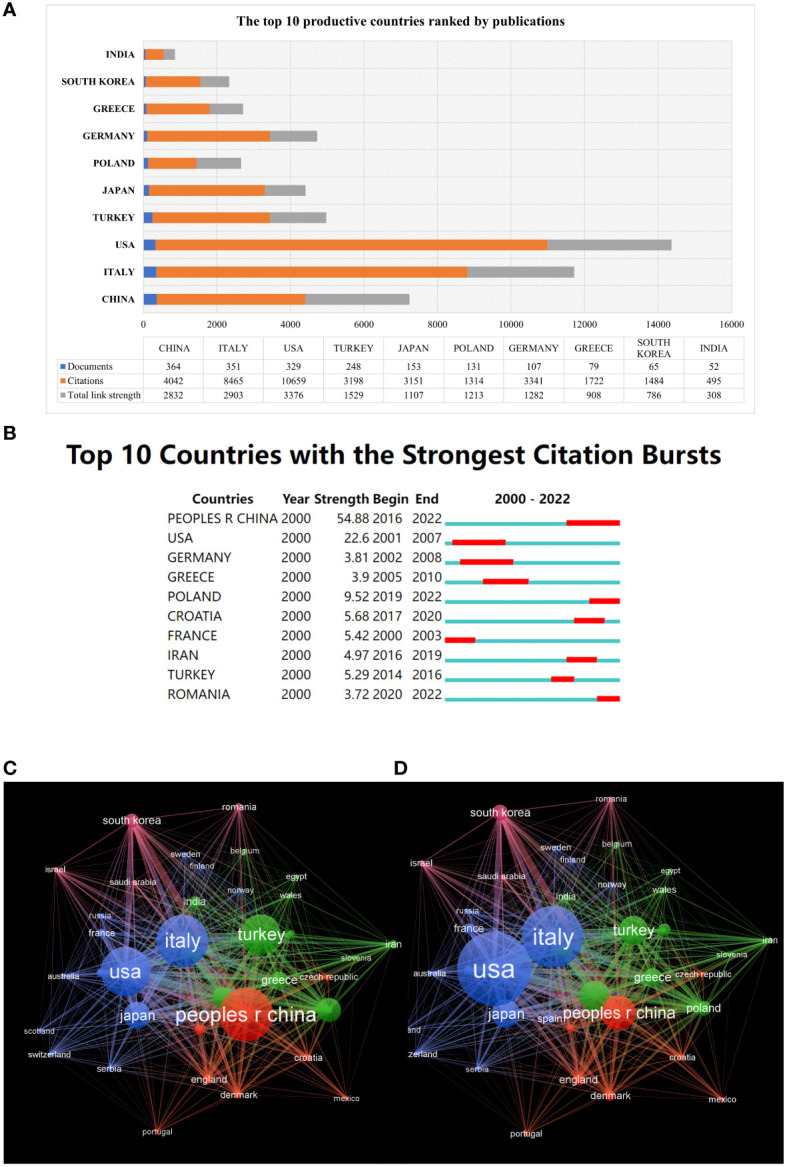
Analysis of countries/regions engaged in AIT. **(A)** The top 10 countries devoted to research concerning AIT. **(B)** The top 10 countries with the strongest citation bursts from 2000 to 2022. **(C)** The cross-country collaboration visualization map. The lines between nodes represent cooperation between countries. Each node represents a country or region. The size of the node is proportional to the number of documents published. **(D)** The cross-country collaboration visualization map. The size of the node is proportional to the number of citations.

According to the VOSviewer analysis, a total of 2290 articles were from 120 countries. As shown in **Figure 4A**, the top 3 countries/regions were China (n = 364, 15.90%), Italy (n = 351, 15.33%) and the United States (n = 329, 14.37%). Strikingly, the top 3 countries contributed 40.85% of the total number of publications. Moreover, as illustrated in **Figure 4B**, each node represents a country or region, and the size of the node is proportional to the number of documents published. The lines between nodes represent cooperation between countries. We find that extensive cooperation between many countries/regions was observed, and the most obvious cooperation was between China and the United States. What is noticeable is that the total link strength of the United States was significantly higher than that of other countries, indicating that it cooperated more closely with other countries. This is also reflected in the fact that the number of documents in China was more than that in the United States, but Chinese citation and total link strength were far less than those of the United States, as shown in **Figure 4C**.

When restricting the timeframe to the last 5 years (2016–2021), the top five most productive countries were China (n=241), Italy (n=113), Turkey (n=82), Poland (n=64) and the United States (n=60).

As shown in the top ten countries/regions with the strongest citation bursts (a significant change in documents in a short period) (**Figure 4D**), China showed the highest burst strength (54.88) since 2017, indicating that there were many scholars studying AIT in China during this period. This has continued up to 2022, suggesting that this field of research will continue to grow.

### Institutions analysis

**Figure 5:**
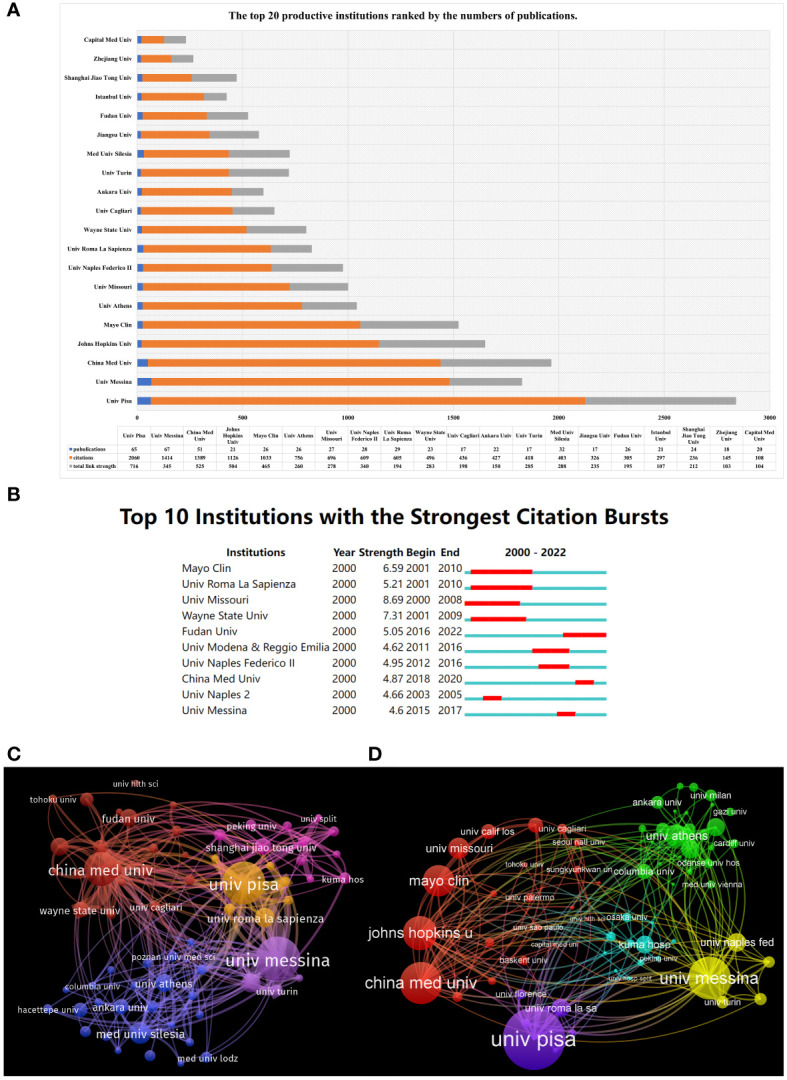
Analysis of institutions engaged in AIT. **(A)** The top 10 institutions devoted to research concerning AIT. **(B)** The top 10 institutions with the strongest citation bursts from 2000 to 2022. **(C)** The visualization map of collaborations between institutions. The lines between nodes represent cooperation between institutions. Each node represents one institution. The size of the node is proportional to the number of documents published. **(D)** The visualization map of collaborations between institutions. The size of the node is proportional to the number of citations.

These documents were contributed by 561 institutions, with the top 20 institutions contributing a total of 577 articles, accounting for 25.20% of the total (**Figure 5A**). As shown in **Figure 5C**, the top 3 institutions/affiliations with the most documents were the University of Messina (n=67), University of Pisa (n = 65) and China Medical University (n = 51). According to the number of citations, the order from highest to lowest was the University of Pisa (citations = 2060), the University of Messina (citations = 1414) and China Medical University (citations = 1389) (**Figure 5D**). Remarkably, six of the top 20 institutions are from China. They are China Medical University, Jiangsu University, Fudan University, Shanghai Jiao Tong University, Zhejiang University and Capital Medical University. The University of Pisa had the highest total link strength of 716, followed by the China Medical University, which had 525. Many Chinese institutions collaborated closely with each other. For burst monitoring of institutions (**Figure 5B**), the top three ranked institutions were University of Missouri, bursting from 2000 to 2008, followed by Wayne State University, bursting from 2001 to 2009, and Mayo Clinic, bursting from 2001 to 2010.

### Journals and co-cited academic journals analysis

**Figure 6:**
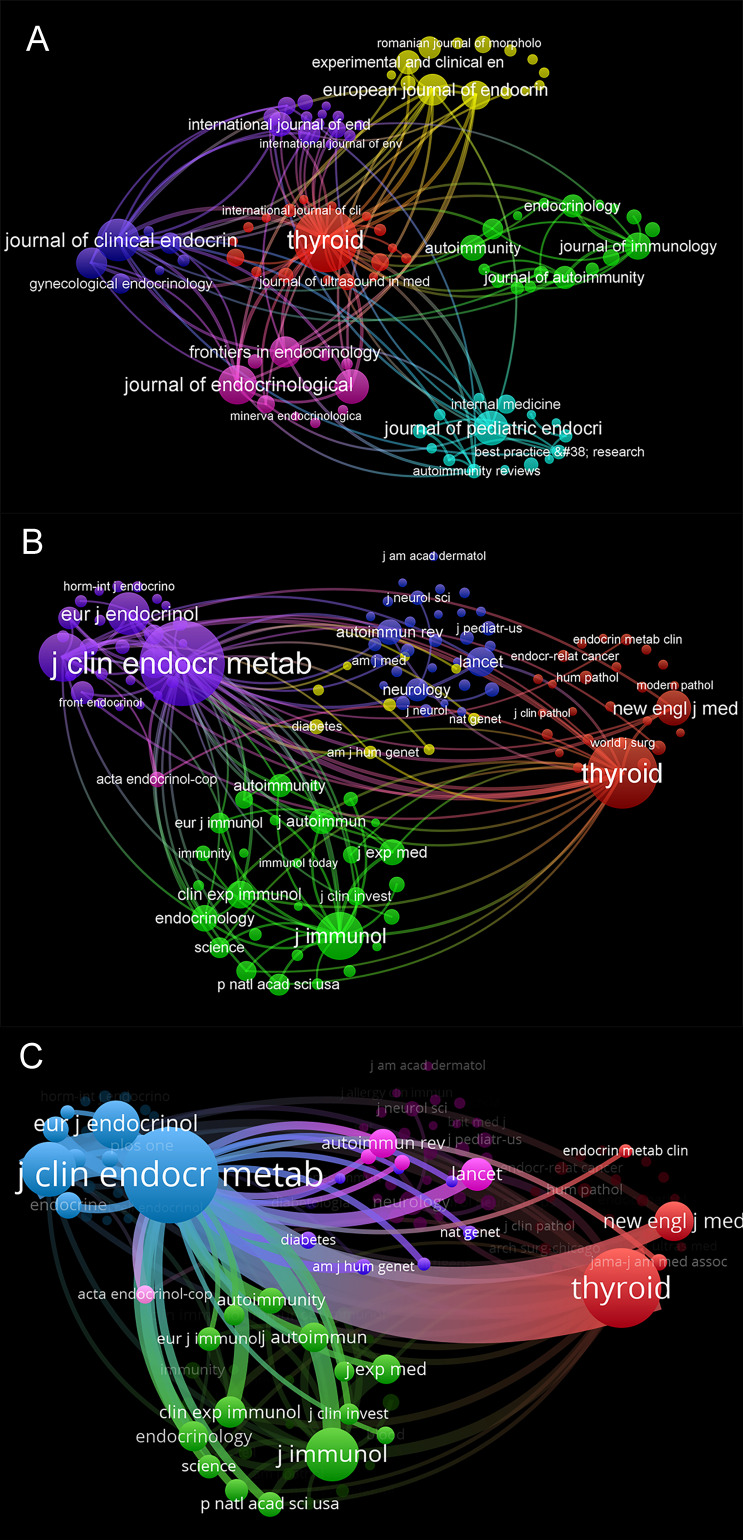
Analysis of journals involved in AIT. **(A)** The visualization map showing academic journals publishing research related to AIT. Each node represents a journal. The size of the node is proportional to the number of documents published. **(B)** The visualization map showing academic journals publishing research related to AIT. The size of the node is proportional to the number of citations. **(C)** The visualization map focused on the total link strength between the *Journal of Clinical Endocrinology and Metabolism* and *Thyroid*.

The WoSCC search showed that the 2290 documents included in the current analysis were published in 723 different journals over the last 23 years since 2000. The top 10 most-cited journals were listed in **Table 2**. As shown in **Figure 6A**, among these journals, *Thyroid* had the highest number of output and impact factors (122, 5.33%, IF=6.568), followed by the *Journal of clinical endocrinology and metabolism* (62, 2.71%, IF=5.958). The influence of journals depends on the number of times they are co-cited, which reflects whether the journal has essential influence in a specific topic (33). Six journals have been cited more than 1000 times. Specifically, the journal with the highest number of citations was the *Journal of Clinical Endocrinology and Metabolism* (5053), followed by *Thyroid* (3721) (**Figure 6B**). We also found that there was a strong link between *Thyroid* and the *Journal of clinical endocrinology and metabolism* (**Figure 6C**). According to the 2022 Journal Citation Reports (JCR), 50% of the top 10 journals were located in the Q1 region. Simultaneously, we noticed that 9 of 10 journals are based in the United States or the United Kingdom (**Table 3**).

**Table 2:** The top 10 journals ranked by number of publications.

Journal	Documents	Percent	Country	Citations	2020 impact factor	2020 JCR partition	H-index
Thyroid	122	5.33%	USA	3751	6.568	Q1	142
Journal of clinical endocrinology & metabolism	62	2.71%	UK	3283	5.958	Q1	353
Journal of endocrinological investigation	53	2.31%	Switzerland	606	4.256	Q2	84
Endocrine	44	1.92%	USA	607	3.633	Q3	81
Journal of pediatric endocrinology	44	1.92%	Germany	481	1.634	Q4	65
European journal of endocrinology	38	1.66%	UK	1699	6.664	Q1	148
Frontiers in endocrinology	37	1.62%	Switzerland	250	5.555	Q1	68
Endocrine journal	37	1.62%	Japan	608	2.349	Q4	72
Clinical endocrinology	32	1.40%	UK	1201	3.478	Q3	353
Journal of immunology	29	1.27%	USA	1049	5.422	Q2	372

**Table 3:** The top 10 co-cited journals ranked by number of citations.

Co-cited journals	Citations	Country	2020 impact factor	2020 JCR partition	H-index
Journal of clinical endocrinology & metabolism	5053	USA	6.568	Q1	353
Thyroid	3721	USA	6.568	Q1	142
Clinical endocrinology	2002	UK	3.478	Q3	353
Journal of immunology	1929	USA	5.422	Q2	372
European journal of endocrinology	1611	UK	6.664	Q1	148
New England journal of medicine	1161	UK	91.253	Q1	1030
Journal of endocrinological investigation	894	Switzerland	4.256	Q2	84
Lancet	856	UK	79.323	Q1	762
Clinical and experimental immunology	783	UK	4.33	Q2	135
Endocrinology	718	UK	4.736	Q2	257

**Figure 7:**
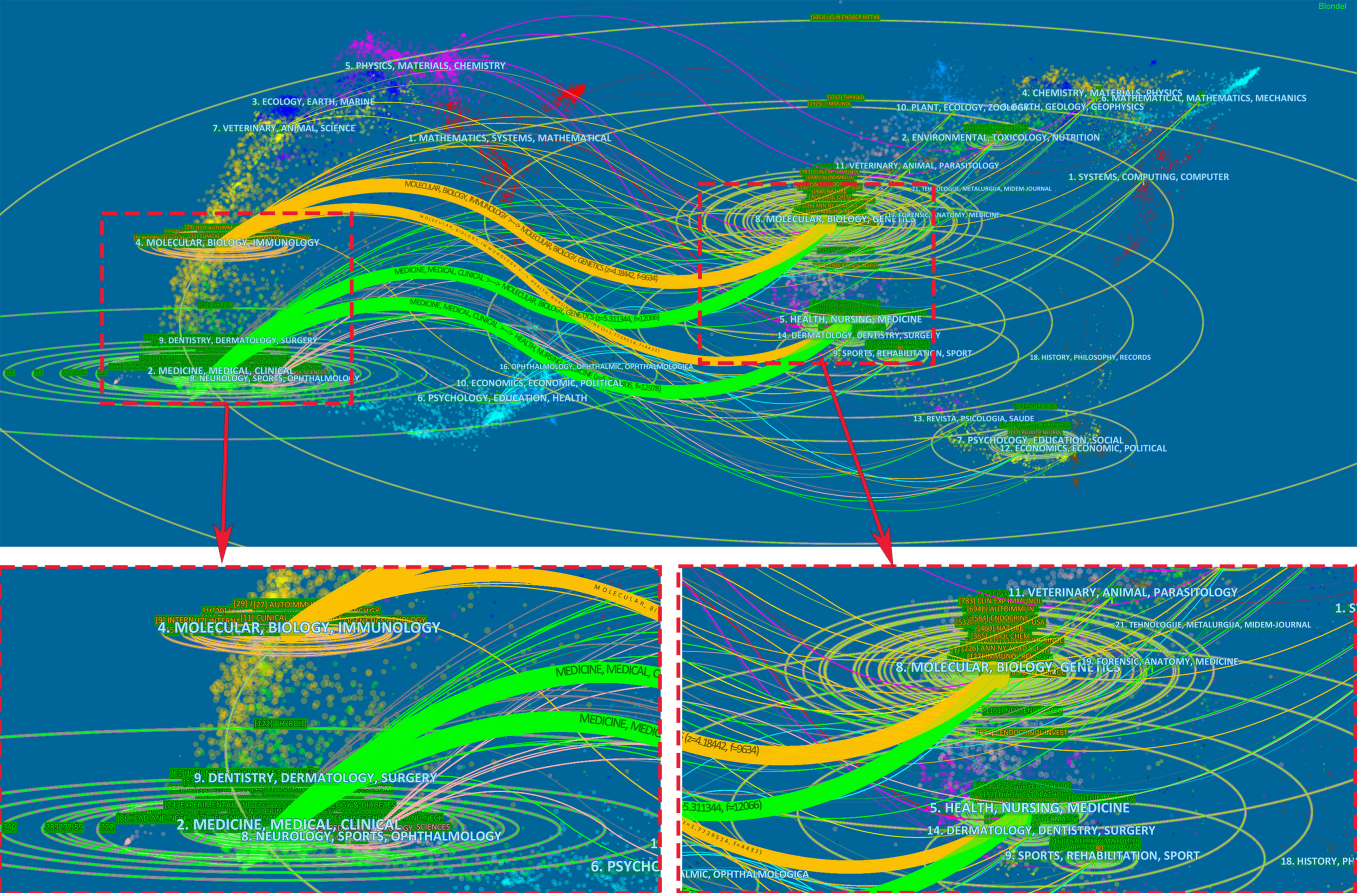
A dual-map overlay of journals related to research on AIT.

The dual-map overlay of journals represents the topic distribution of academic journals (31) (**Figure 7**). The citing journals are located on the left, while the cited journals are on the right, and the colored paths illustrate the citation relationships (39). As shown, the yellow and green paths indicate that studies published in health/nursing/medicine or molecular/biology/genetics journals are usually cited in the studies published in medicine/medical/clinical or molecular/biology/immunology journals.

### Hotspots and Frontiers analysis

**Figure 8:**
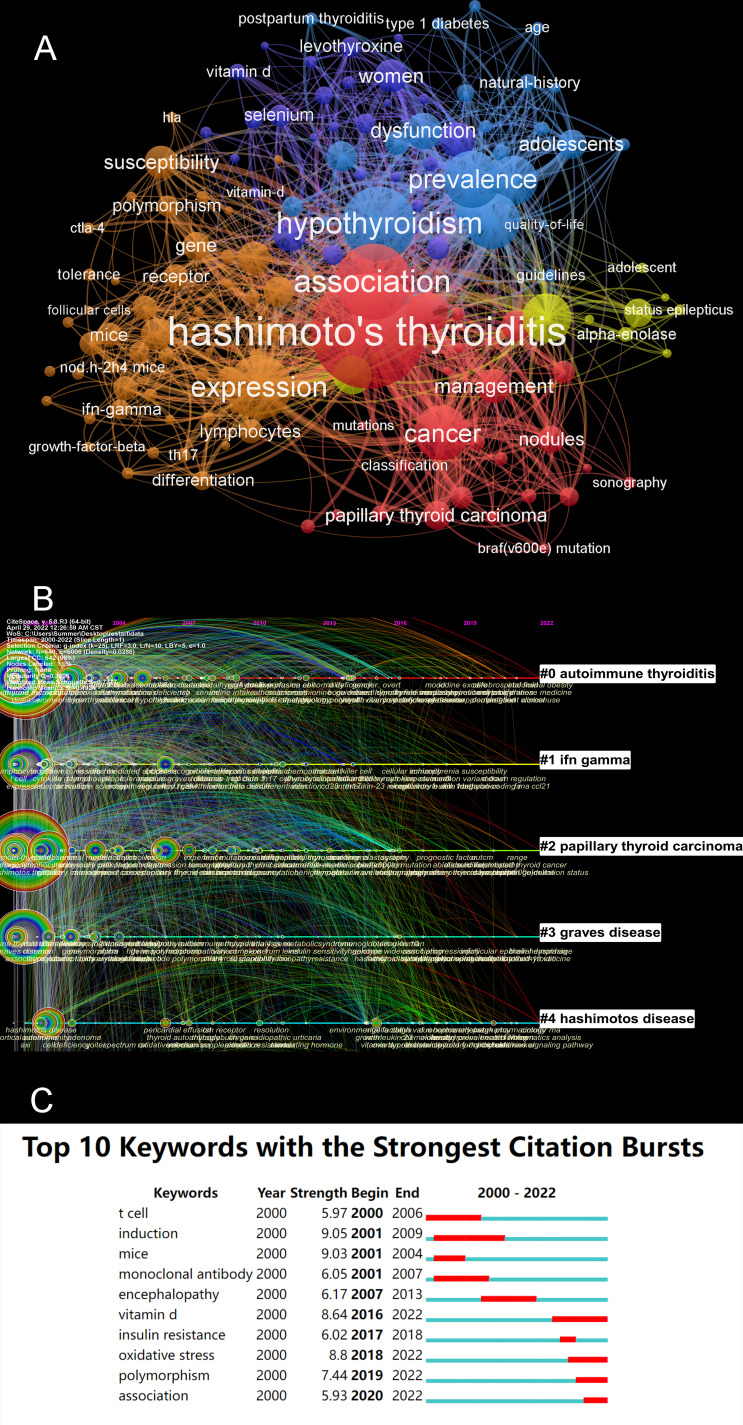
**(A)** A network map of keywords. **(B)** The timeline view of keywords. **(C)** The keywords with the strongest citation bursts in articles related to AIT research.

We used VOSviewer to construct a network map of keywords (**Figure 8A**). Excluding hashimoto’s thyroiditis (574) and some unmeaningful words, the keywords appearing at high frequency among these documents were hypothyroidism (299), expression (242), prevalence (203), antibodies (195), cancer (191), diagnosis (162), pathogenesis (114), dysfunction (104), iodine (89), susceptibility (89), T-cells (85) and so on. These keywords have been used in research in previous studies or are the current focus of research. **Figure 8A** shows the blue, purple, red, orange and yellow clusters, indicating five research categories. The blue clusters included the keywords hypothyroidism, dysfunction, prevalence, age, adolescence, natural history and so on. The purple cluster included the keywords levothyroxine, selenium, vitamin D, women, postpartum thyroiditis and type 1 diabetes. The red clusters included the keywords hashimoto’s thyroiditis, management, classification, nodules, cancer and so on. The orange clusters included the keywords mice, Th 17, IFN-gamma, growth-factor beta, gene, susceptibility, polymorphism, tolerance T-cells, regulatory T-cells, differentiation, genes, receptor, expression, susceptibility and so on. The last set of clusters, the yellow ones, included the keywords guidelines, quality of life and so on. We also used CiteSpace to cluster keywords chronologically, and we showed the largest cluster in **Figure 8B**. They were “autoimmune thyroiditis”, “IFN-γ”, “papillary thyroid cancer”, “Graves’ disease” and “Hashimoto disease”.

CiteSpace was also used to illustrate the keywords with strong citation bursts. As **Figure 8C** demonstrates, we screened out top four keywords whose citation bursts continued to 2022, including “Vitamin D”, “oxidative stress”, “polymorphism” and “association”.

## Discussion

### General information

In this era of information explosion, it is comparatively more difficult to grasp the emphasis in a specific field and accurately obtain cutting-edge information as well as identify the research trend and hotspots. Knowledge management and bibliometric analysis are often used as methods to address the concerns of scientists (40–42). In this article, we aimed to employ an innovative method to analyze and visualize the knowledge structures of the AIT research field by employing software such as CiteSpace and VOSviewer, which are bibliometric tools. In the present study, we performed a systematic literature search of the Web of Science databases for articles published in the last two decades about AIT (2000–2022). After excluding studies that did not meet the screening criteria, this scientometric study included 2290 English papers published in 723 journals with 39661 co-cited references from 561 institutions in 120 countries/regions. Among 28 RCTs, 8 have focused on the effect of selenium supplementation on autoimmune thyroiditis (43–46), and 7 meta-analyses were conducted on this contradictory topic (38, 47, 48). The effect of levothyroxine and other nutrients such as vitamin D on thyroid function has also been studied in many documents (49–53). Also, meta-analyses have also summarized the relationship between autoimmune thyroiditis and other diseases such as chronic urticaria (54), systemic lupus erythematosus (55), thyroid cancer (36, 56–58) as well as depression and anxiety (59).

Based on the results of reference analysis and keyword extraction, those dominating were “apoptosis”, “insulin resistance”, “encephalopathy”, “IFN-γ” and others mentioned in the top-ranked items may have been popular research topics over the past years but may have slowed down with the development of other novel directions such as “papillary thyroid cancer”, “Vitamin D”, “oxidative stress”, “polymorphism” and “association”. Through the analysis of references and keywords, we can understand the current research status of the field and scientifically predict the future research trends to provide scientific researchers with the direction to move forward. In the following sections we will specify the current research status of the first four keywords.

### Research hotspots

#### Papillary thyroid cancer and AIT

Papillary thyroid carcinoma (PTC) is the most common type of thyroid neoplasm and accounts for 80-90% of all thyroid cancers. The association between HT and papillary thyroid carcinoma (PTC) has been hotly debated in recent years. Several studies have found a relationship between HT and PTC (60–62). However, based on 8 fine-needle aspiration (FNA) studies and 8 thyroidectomy studies, Jankovic et al. found that there was no statistically significant correlation between HT and PTC (37). Two meta-analyses reported that HT predisposed patients to the development of PTC (58, 63). The underlying mechanism of developing malignancies in HT patients is not clear. This may be because the accumulation of excess ROS in the thyroid gland can cause DNA damage, resulting in DNA damage and mutations that finally cause the development of PTC (64), or increased levels of TSH can also stimulate thyroid tissue epithelial proliferation. Zhang et al. reported that lncRNAs and mRNAs play an important role in establishing the different clinical characteristics between patients with PTC/HT(+) and patients with PTC/HT (–), which might provide new insights to further understand the correlation between PTC and HT (65).

#### Oxidative stress and AIT

Oxidative stress (OS), resulting from an imbalance between the excessive production of reactive oxygen species (ROS) and a reduction in antioxidant production (66). To date, research on HT and ROS has mainly progressed on two fronts: one is that oxidative stress may lead to the development and exacerbation of HT (67, 68); at the same time, some biomarkers of oxidative stress may present a potential diagnostic value (69); the other is that patients with Hashimoto thyroiditis show enhanced oxidative stress. Many researchers have proven this hypothesis, and they found that some of the oxidative stress markers were elevated, such as serum levels of advanced glycation end products (AGEs), malondialdehyde (MDA) and derived reactive oxygen metabolites (d-ROMs), while many others were decreased, including soluble receptor (sRAGE), serum glutathione (GSH), total antioxidant capacity (TAC) and biological antioxidant potential (BAP) (70–73).

In terms of prevention and treatment, Rosaria Maddalena Ruggeri et al. (74) showed that low intake in animal foods had a protective effect against thyroid autoimmunity and showed the positive influence of such nutritional patterns on redox balance and potentially on oxidative stress-related disorders. Ihsan Ates et al. (75) reported that levothyroxine replacement decreases oxidant status and increases antioxidant status following 6 months of levothyroxine replacement. Some researchers have also found that selenium supplementation may decrease oxidative stress in HT patients by decreasing thyroid peroxidase antibody titers (76–78).

In summary, there is still much work to be done on Hashimoto’s thyroiditis and oxidative stress, and more randomized clinical trials are needed to clearly confirm the cause and effect as well as elucidate the mechanisms at a deeper level.

#### Vitamin D and AIT

Vitamin D, a steroid hormone whose main role is to control mineral homeostasis, was recently found to exert a number of extra-skeletal effects, such as endocrine effects on cells of the immune system, producing anti-inflammatory and immunomodulatory functions (79, 80).

We summarize that the current studies concerning vitamin D and HT primarily include two aspects. First, what is the relationship between HT and vitamin D? Guanqun Chao et al. concluded that TSH is negatively correlated with 25(OH)D levels, while FT3 and FT4 levels were positively correlated with 25(OH)D levels. Several studies have found association between TSH, TPOAb, TgAb and vitamin D in women (81–83). However, some other researchers declared that there was no association between vitamin D and HT (84, 85). Current data on the role of vitamin D in HT remain controversial because most of the studies are cross-sectional surveys with a small number of subjects, which are easily affected by the heterogeneity of the study population, seasonal variation in blood sampling, different definitions of vitamin D deficiency, and other factors (86).

Second, we examined whether vitamin D supplementation influenced HT. A nationwide, randomized, double-blind, placebo-controlled trial demonstrated that vitamin D supplementation for five years lowered the rate of autoimmune disease by 22% (87). Su Wang et al. (49) suggested that vitamin D supplementation could diminish the serum TPO-Ab and Tg-Ab titers of patients with HT in the short term (approximately six months). R Krysiak et al. (88, 89) also noticed that vitamin D supplementation was able to reduce TPO-Ab titers and suggested that women with PPT may benefit from l-thyroxine treatment. However, this suggestion was strongly criticized in a letter from the same journal, and they explained that more studies are required to definitively confirm that vitamin D can offer preventive and therapeutic benefits across a wide range of physiological states and chronic nonskeletal disorders (90).

In summary, as far as the literature is concerned, vitamin supplements are of great benefit for HT to some degree. However, there are still some reports contradicting this viewpoint, making it difficult to establish a consistent conclusion. Vitamin D metabolism may also exhibit racial differences, as studies have reported, to some degree explain these opposite conclusions (91). To better understand the role of vitamin D in preventing and improving HT, several and more randomized, controlled, prospective studies are needed to demonstrate the causality of vitamin D in HT and accordingly provide information on the dose and timing of supplementation.

#### Polymorphism and AIT

Human gene polymorphisms play a crucial role in elucidating the susceptibility and tolerance of humans to disease and explaining the clinical phenotypic diversity as well as the various responses to drug therapy.

AITD susceptibility genes can be divided into thyroid-specific (TG, TSHR) or immunomodulatory (FOXP3, CD25, CD40, CTLA-4, HLA or others) genes; among them, Foxp3 and CD25 play a key role in establishing peripheral tolerance, while CD40, CTLA-4 and HLA genes are critical for T lymphocyte activation and antigen presentation (92). Emerging evidence suggests that single-nucleotide polymorphisms (SNPs) in immunoregulatory genes may functionally impede the normal development of central and peripheral tolerance and alter the interaction of T cells with antigen-presenting cells (APCs) in the immune synapse. Numerous studies have found that gene polymorphisms, such as CTLA4 (93–95), TNF -308 (96), TRAF1 (97), microRNA (98), NFKB1 (99), NLRP1 (100), ICAM-1 (101), ZFAT (102) and so on, are significantly associated with AITD. Racial variations in AITD prevalence further accentuate potential genetic polymorphisms and the role of genetic susceptibility in the etiology of AIT (103). A variety of polymorphisms suggest that the genetic triggers of autoimmunity may be due to inherited abnormalities ranging from central tolerance of the thymus and peripheral tolerance of Tregs to appropriate costimulation of T cells and APC in the immune synapses (92).

In summary, many studies have focused on the gene levels and made great contributions to the mechanism of HT diagnosis and prevention. However, A Jabrocka-Hybel et al. (104) reported that even when using multivariate models to analyze genetic regions most commonly and significantly associated with AITD, it was not possible to predict risk of developing HT. Therefore, the strong association between a single genetic region and HT must be interpreted cautiously, and to reveal the intricacies of genetic associations with HT, it is necessary to study multiple factors simultaneously.

### Strengths and limitations

An analysis based on bibliometric tools such as CiteSpace and VOSviewer provides a better illustration of evolving research priorities and trends, as well as relatively comprehensive and objective data analysis, compared to that of a traditional overview. However, there are some limitations in the research design. First, the publications we included in the analysis had a cut-off date of March 20, 2022, but the WOS Core Collection data continues to be updated and some of the 2022 literature is already online, which was not included in our work so that our paper does not fully reflect the reality of 2022. Second, only English documents were included; thus, contributions in other languages may be ignored. Third, because of the format requirement of CiteSpace, only publications in the WoSCC database were finally included in our statistics, which may ignore articles that are only in other databases, such as PubMed, Medline and Scopus. However, because of the significant cross-replication of the literature in the various databases and the authority of the WoSCC database, we consider that this work still can be applied to present the overall situation and general trend for this field.

## Conclusion

Our study is the first bibliometric analysis employing software including CiteSpace, VOSviewer and Pajek to study the research trends and hotspots of AIT research, and the results provide an updated analysis of the global scientific output related to AIT research from 2000 to 2022. The number of publications in the field of AIT has increased year by year. The current research focused on papillary thyroid cancer, oxidative stress, vitamin D, polymorphisms and so on. Our study illustrated basic scientific knowledge and various interrelationships concerning HT and also provided essential clues on research trends and frontiers. It is our hope that this study will help researchers to better grasp current overall trends in this field.

## Data availability statement

The original contributions presented in the study are included in the article/**Supplementary Material**. Further inquiries can be directed to the corresponding author.

## Author contributions

QL and JL conceived the study. WY participated in the statistical analysis. QL drafted the article. JL revised the article. All of the authors read and approved the final version of the manuscript.

## Funding

This study was supported by the Chinese National Natural Science Foundation (grants 82100831).

## Conflict of interest

The authors declare that the research was conducted in the absence of any commercial or financial relationships that could be construed as a potential conflict of interest.

## Publisher’s note

All claims expressed in this article are solely those of the authors and do not necessarily represent those of their affiliated organizations, or those of the publisher, the editors and the reviewers. Any product that may be evaluated in this article, or claim that may be made by its manufacturer, is not guaranteed or endorsed by the publisher.

## Supplementary material

Supplement 1 Flowchart for the selection of documents and the process of literature analysis in the field of AIT.
