# Supplementary figures and images for: Emerging trends and hot spots in autoimmune thyroiditis research from 2000 to 2022: A bibliometric analysis
Source: Front Immunol. 2022 Aug 11;13:953465. doi: 10.3389/fimmu.2022.953465 (PMC9402901; doi:10.3389/fimmu.2022.953465)

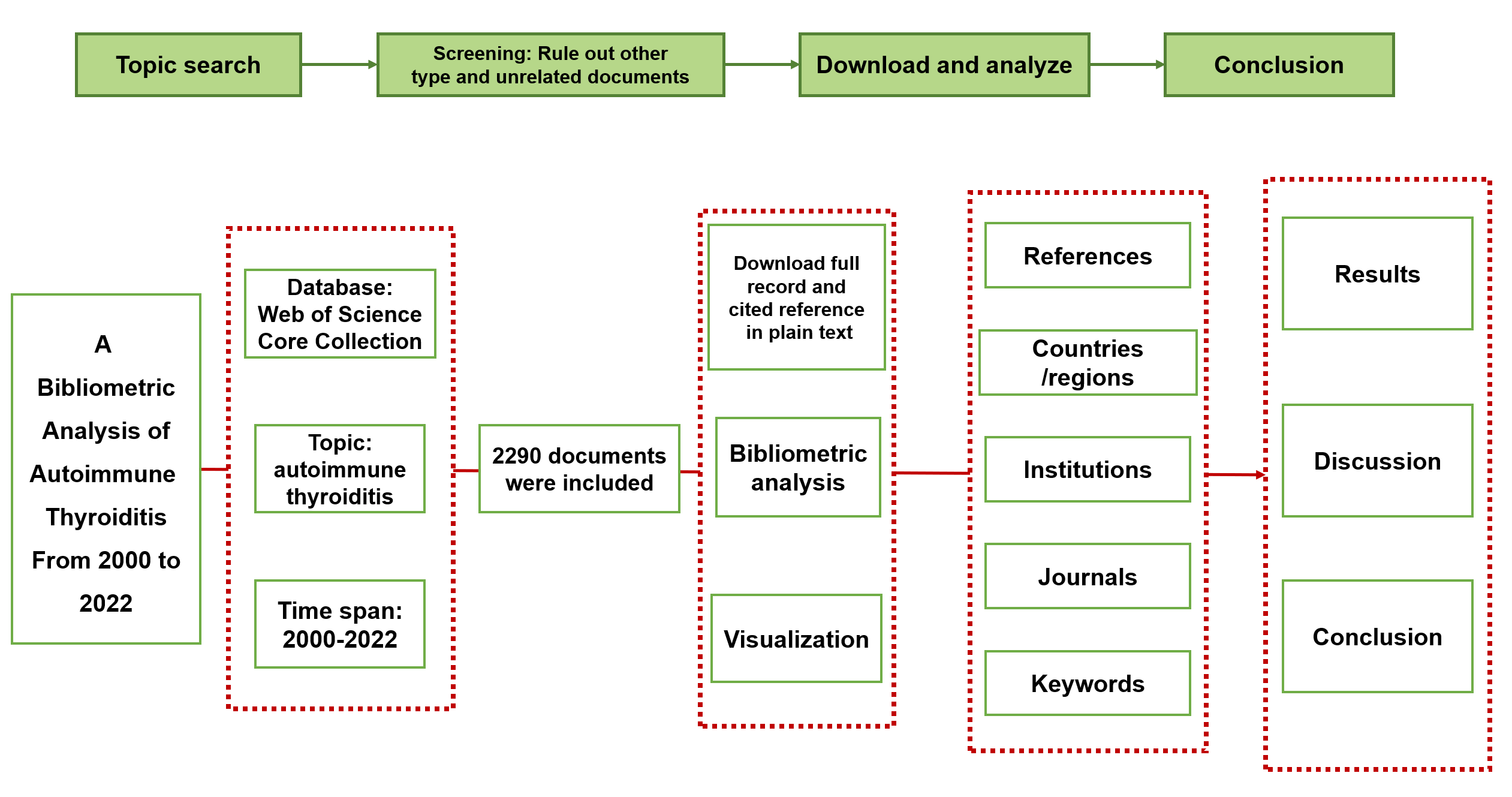

Supplement: Supplement 1 — Flowchart for the selection of documents and the process of literature analysis in the field of AIT. [file Image_1.png]
